# Acculturation and Health Status in the Children’s Healthy Living Program in the Pacific Region

**DOI:** 10.3390/ijerph21040448

**Published:** 2024-04-06

**Authors:** Kalanikiekie S. Sparks, Marie K. Fialkowski, Rica Dela Cruz, Andrew Grandinetti, Lynne Wilkens, Jinan C. Banna, Andrea Bersamin, Yvette Paulino, Tanisha Aflague, Patricia Coleman, Jonathan Deenik, Travis Fleming, Rachel Novotny

**Affiliations:** 1 Public Health Division, Acute and Communicable Disease Section, Oregon Health Authority, Salem, OR 97301, USA; kalanikiekie.s.sparks@oha.oregon.gov; 2 Nutrition Support Shared Resource, University of Hawaiʻi Cancer Center, University of Hawaiʻi at Mānoa, Honolulu, HI 96813, USA; 3 Human Nutrition, Food and Animal Sciences Department, College of Tropical Agriculture and Human Resources, University of Hawaiʻi at Mānoa, Honolulu, HI 96822, USA; ricadc@hawaii.edu (R.D.C.); jcbanna@hawaii.edu (J.C.B.); novotny@hawaii.edu (R.N.); 4 Office of Public Health Studies, University of Hawaiʻi at Mānoa, Honolulu, HI 96822, USA; andrewg@hawaii.edu; 5 Biostatistics Shared Resource, University of Hawaiʻi Cancer Center, University of Hawaiʻi at Mānoa, Honolulu, HI 96813, USA; lynne@cc.hawaii.edu; 6 Department of Biology and Wildlife, College of Natural Sciences and Mathematics, University of Alaska Fairbanks, Fairbanks, AK 99775, USA; abersamin@alaska.edu; 7 Margaret Perez Hattori-Uchima School of Health, University of Guam, Mangilao, GU 96913, USA; paulinoy@triton.uog.edu; 8 Cooperative Extension and Outreach, College of Natural and Applied Sciences, University of Guam, Mangilao, GU 96913, USA; taflague@triton.uog.edu; 9 Cooperative Research, Extension, and Education Services, Northern Marianas College, Saipan, MP 96950, USA; patricia.coleman@marianas.edu; 10 Tropical Plant and Social Sciences Department, College of Tropical Agriculture and Human Resources, University of Hawaiʻi at Mānoa, Honolulu, HI 96822, USA; jdeenik@hawaii.edu; 11 Agriculture, Community and Natural Resources Division, American Samoa Community College, Pago Pago, AS 96799, USA; travis@rocketmail.com

**Keywords:** acanthosis nigricans, acculturation, body mass index, child, obesity, overweight, U.S.-Affiliated Pacific, waist circumference

## Abstract

Acculturation/enculturation has been found to impact childhood health and obesity status. The objective of this study is to use cross-sectional data to examine the association between proxies of adult/caregiver acculturation/enculturation and child health status (Body Mass Index [BMI], waist circumference [WC], and acanthosis nigricans [AN]) in the U.S.-Affiliated Pacific Islands (USAPI), Alaska, and Hawaiʻi. Study participants were from the Children’s Healthy Living (CHL) Program, an environmental intervention trial and obesity prevalence survey. Anthropometric data from 2–8 year olds and parent/caregiver questionnaires were used in this analysis. The results of this study (n = 4121) saw that those parents/caregivers who identified as traditional had children who were protected against overweight/obesity (OWOB) status and WC > 75th percentile (compared to the integrated culture identity) when adjusted for significant variables from the descriptive analysis. AN did not have a significant association with cultural classification. Future interventions in the USAPI, Alaska, and Hawaiʻi may want to focus efforts on parents/caregivers who associated with an integrated cultural group as an opportunity to improve health and reduce child OWOB prevalence.

## 1. Introduction

Parent and child acculturation/enculturation have impacts on childhood health and weight status [1]. This is a major public health concern as childhood obesity prevalence and its physical and mental health complications can carry on into adulthood [2,3,4]. In 2010, the Pacific Island Health Officers Association (PIHOA) declared a regional health emergency in the underserved U.S.-Affiliated Pacific Islands (USAPI) due to the rise in non-communicable diseases like cancer, cardiovascular disease, and diabetes [5]. The USAPI consist of three territories: American Samoa, Commonwealth of the Northern Mariana Islands [CNMI], and Guam. In addition, there are three freely associated states: the Federated States of Micronesia [FSM], Republic of Palau, and the Republic of the Marshall Islands [RMI]. The U.S. has responsibility for the health operations of these six jurisdictions. Public health concerns relating to the rise in non-communicable diseases can be tied to changes in adult and child nutrition and weight status [6].

The National Health and Nutrition Examination Survey (NHANES) lacks surveillance data on adult and childhood obesity in the USAPI in addition to Alaska and Hawaiʻi [7]. The lack of comprehensive NHANES data on childhood obesity and its risk factors in this region is problematic when needing to formulate health interventions to combat childhood obesity. Addressing this issue requires specific and culturally appropriate interventions due to cultural differences (e.g., consumption of traditional foods, family involvement, ethnic disparities in health) and historical trauma (i.e., perceived racism) [8]. Prior to Western contact, indigenous people of the Pacific followed diets that consisted of traditional foods that were grown from the land and found in the sea [9]. These traditional diets have been shown to protect health and provide opportunities for the exposure to and intake of healthful foods for Pacific peoples. Western contact introduced new foods and lifestyle changes that have been incorporated into Pacific traditional diets and cultures, leaving it unclear as to what is traditional or acculturated today [10].

The relationship between culture (acculturation vs. enculturation) and indicators of health is established in the literature [11,12,13]. In most migrant populations in the U.S., acculturation is associated with an increased risk of overweight status and obesity in children [14,15,16]. However, most of this research has been conducted in ethnic groups (e.g., Hispanic Mexican, Asian Hmong) that are not representative of the USAPI, Alaska, and Hawaiʻi region.

Information on the relationship among culture, health, and OWOB is limited in the remote jurisdictions of the USAPI, Alaska, and Hawaiʻi [17,18]. The ethnic diversity, unique history, and varied political relationships these jurisdictions have with the U.S. may have a significant impact on the relationship between culture and health in these underserved indigenous groups [5]. Evidence suggests there is a great disparity in the prevalence of childhood obesity among certain ethnic minorities in the Pacific region [8,17]. The purpose of this paper is to use cross-sectional data collected by the Children’s Healthy Living (CHL) Program for Remote Underserved Minority Populations in the Pacific Region to examine associations between proxies of adult/caregiver acculturation/enculturation and child health status (Body Mass Index [BMI], waist circumference [WC], and acanthosis nigricans [AN]) in the USAPI, Alaska, and Hawaiʻi [19]. The main hypothesis is those parents or caretakers who report following a more acculturated lifestyle (e.g., a more Western lifestyle), established by a cultural identity subscale score, will have children with higher BMI scores (overweight/obesity (OWOB) > 85th percentile), higher WC (>75th or 90th percentile), and/or signs of AN.

## 2. Materials and Methods

The Children’s Healthy Living (CHL) Program community randomized trial was a 2-year environmental intervention trial with a mission “to evaluate the capacity of the region to build and sustain a healthy food and physical environment to help maintain healthy weight and prevent obesity among young children in the Pacific region” [19]. Five jurisdictions (Alaska, American Samoa, CNMI, Guam, and Hawaiʻi) participated in the trial. In addition, a childhood obesity prevalence survey was conducted in the Freely Associated States of Micronesia (FAS), which includes FSM, Palau, and RMI. The FAS did not participate in the intervention trial. Participants in the present study were children between 2 and 8 years of age living in one of the selected jurisdictions. For the intervention jurisdictions, 18 communities (9 intervention and 9 control) were selected to evaluate the effectiveness of the program [19]. Communities were chosen based on specific criteria related to population size, proportion of indigenous/native descent, and proportion under 10 years of age. Other criteria included adequate settings for sampling children and community cohesiveness and accessibility (see Wilken[s] et al., 2013 [19] for more detailed information on process of community selection criteria). Recruitment sites operated by CHL staff members targeted school and community venues (e.g., Head Starts, pre-schools/daycares, Special Supplemental Nutrition Program for Women, Infants and Children [WIC] sites). The recruitment goal per community was 180 children. Data were collected from October 2012 to February 2014 for the 5 environmental intervention trial jurisdictions. For FAS, the prevalence survey was intended to provide representative samples with 200 children each for RMI, Palau, and the four FSM jurisdictions (Yap, Chuuk, Pohnpei, Kosrae), all of which have >25% indigenous/native population and >10% under 10 years of age. The main population centers/islands for each jurisdiction were divided into geographic sectors with representation sampling based on the country-specific censuses. Data were collected from October 2013 to May 2015 for the FAS prevalence jurisdictions. The CHL Program was funded by the U.S. Department of Agriculture (USDA), Agriculture and Food Research Initiative Grant. Institutional Review Board (IRB) approvals from the University of Alaska Fairbanks, University of Guam, the University of Hawaiʻi at Mānoa and in Palau were attained prior to data collection. The American Samoa Community College, the Northern Marianas College, and the institutional partners in the Freely Associated States in Micronesia ceded to the University of Hawaiʻi at Mānoa IRB. A signed consent form was obtained from parents/caregivers who had children participating in the CHL Program. Additionally, children provided their assent for measurements. For the purposes of this analysis, a cross-sectional data set was created from the baseline measurements of the intervention trial and the FAS prevalence survey.

### 2.1. Anthropometry

All staff measuring anthropometry in the selected jurisdictions were trained to collect standardized data to ensure accuracy and reliability [20]. Trained staff collected height, weight, and WC using standardized measurement equipment. Portable stadiometers were used for height in centimeters (Perspective Enterprises, PE-AIM-101; Portage, MI, USA) and portable scales (Seca, Model 876; Chino, CA, USA) were used for weight measurements in kilograms. Children were measured in lightweight clothing with no shoes and bulky hair bands removed. WC was measured at the umbilicus to the nearest 0.1 cm with a plastic measuring tape [21] (Seca, Model 201; Chino, CA, USA). The protocol for measurement collection involved taking 3 measurements at each visit and ensuring that two of the three readings were within 0.2 units of each other. If the readings were not within 0.2 units, all 3 measurements were repeated. The mean height, weight, and WC measurements were then calculated for each participant [19].

Body Mass Index (BMI) is an indication of body composition that can then be associated with possible disease risk. BMI was calculated by dividing the child’s weight (kilograms) by the square of the child’s height (meters) using 2000 CDC growth charts for ages 2 to <20 years [22]. The criteria for establishing overweight and obesity status in children are different than in adults to account for growth and use age- and sex-specific percentiles of the CDC reference for BMI. Children’s height (cm) and weight (kg) were analyzed using SAS code for the 2000 CDC Growth Charts to determine sex-specific BMI-for-age, and children with a BMI less than the fifth percentile were classified as “Underweight”, 5th to 84th percentiles as “Healthy”, 85th to 94th percentiles as “Overweight”, and those at or above the 95th percentile as “Obese” [23]. For purposes of this study, BMI was further dichotomized into Healthy weight and OWOB (Overweight/Obese). Underweight were not included in the analysis. Although dichotomized values can lead to misleading results [24], it was useful in this analysis since the variables were on approximately the same scale, so the strength of association could be interpreted as a proportional increase or decrease of child weight status.

WC is also an important emerging indicator of health and is used as a predictive marker for diabetes and cardiovascular disease and metabolic syndrome in children [25]. Two age- and sex-specific cut-off points were used for WC in this analysis [26]. The first cut-off point used as a measurement for health monitoring and diagnostics was <90th percentile and ≥90th percentile [26]. Those children with a WC ≥ 90th percentile have been shown to have more risk factors than those below this level [25]. When looking at the U.S. anthropometric reference data for children and adults [26], the 75th percentile [27] was also used to see those children in these populations with higher abdominal obesity in comparison to those in the 95th percentile. As there are no set standards for children currently in use, both WC ≥ 75th and ≥90th percentile groups were seen as potential cut-off points for overweight status and obesity.

### 2.2. Acanthosis Nigricans (AN)

Presence of AN, a sign of pre-diabetes in adults and most commonly seen in children who are obese [28,29], was measured by assessing the back of the children’s necks in plain sight (hair and shirt collars moved) for color and texture using Burke’s quantitative scale of severity [30]. A score corresponding to severity of either 0 = absent; 1 = present [clearly present on close visual inspection, not visible to the casual observer]; 2 = mild [limited to the base of the skull, does not extend to the lateral margins of the neck]; 3 = moderate [extending to the lateral margins of the neck, usually 3–6 inches]; or 4 = severe [extending anteriorly, >6 inches] was assigned and documented. Those children who screened positive for AN were referred to a health care provider [19]. For purposes of this study, those with a report of one or more were considered to have AN and were compared to those who did not (score of 0).

### 2.3. Cultural Identity Subscale Score (Acculturation/Enculturation)

The cultural identity of the parent/caregiver was determined through an adapted questionnaire to allow for the evaluation of acculturation/enculturation [31]. This questionnaire was divided into 2 subscales: a 4-item ethnic traditional cultural identity subscale and a 4-item U.S. mainland cultural identity subscale. These questions assessed the traditional and U.S. mainland culture, the personal feelings, knowledge of practices, association with others, and impact the culture had on their lifestyle. A 5-point subscale was used in each domain: 1 [very knowledgeable; very involved; very positive; most of the time] to 5 [not at all knowledgeable; not at all involved; very negative; not at all associated]. Total scores were summed from each subscale and categorized into one of four acculturation categories: integrated, with high affiliation with traditional and U.S. mainland identities; traditional, high affiliation with traditional identity only; assimilated, high affiliation with U.S. mainland identity only; and marginalized, low affiliation with ethnic and U.S. mainland identity [19]. Only those with complete information for all 8 questions were analyzed. Assimilated was considered the group most associated with the U.S. mainland/Western lifestyle as defined in the hypothesis. Integrated was used as the comparison group in the statistical model.

### 2.4. Socio-Demographic and Household Characteristics

Socio-demographic characteristics of the child and family were reported by a parent or caregiver on a standardized form [19]. Questions pertaining to the parent/caregiver included education, income, and whether they resided in a multigenerational household. Multigenerational was defined as having more than two generations living in the household. Education and income were dichotomized for this analysis. Due to the large number of missing values for income (n = 929), missing values were included as a separate income category (“not reported”) in analyses. Due to the small numbers in each of the many ethnic groups, race/ethnicity was defined by the Office of Management and Budget (OMB) classifications for inclusion in the statistical models: American Indian/Alaska Native (AIAN) or Black, Asian, Native Hawaiian/Pacific Islander (NHPI), more than one race, and White. A variable for indigenous ethnic groups was developed from the Pacific Islander and Alaska Native ethnicities, indicating ethnicity matching the jurisdiction in which the data were collected. Indigenous groups of each jurisdiction include Palauan in Palau, Yapese in Yap, CHamoru/Chamorro in Guam, CHamoru/Chamorro and Carolinian in CNMI, Chuukese in Chuuk, Pohnpeian in Pohnpei, Kosraean in Kosrae, Marshallese in RMI, Samoan in American Samoa, Native Hawaiian in Hawaiʻi, and Alaska Native in Alaska. Questions pertaining to the child included sex, age, race, and ethnic background.

### 2.5. Statistical Analysis

All statistical analyses were performed using SAS 9.4 (SAS Institute Inc., Cary, NC, USA) [32]. Descriptive summary statistics were performed (frequencies, percentages, means, and standard deviations as appropriate) for all variables. Chi-square tests or *t* tests were calculated to test the differences between each possible acculturation category and OWOB (healthy weight and OWOB), high WC, and presence of AN. The Underweight category was excluded from the analysis. Variables that showed a chi-square or *t* test *p*-value < 0.15 in the cross tabulations were then included in the logistic regression model. A cut-off *p*-value of 0.05 can falsely eliminate variables that would be important in the preliminary stages of analysis [33]. Logistic regression models analyzed the association of culture identity score with BMI status, WC in the 75th percentile, WC in the 90th percentile, or AN and adjusted for significant variables from descriptive analysis; in addition, the model included jurisdiction as a strata variable and community as a random effect to account for the clustering of participants in communities within jurisdictions. AN was left out of the final model due to its non-significance in the inferential statistics and crude model. Odds ratios with significant values used for 95% confidence intervals did not include the null value of 1.

## 3. Results

Those included for this study provided complete information on the following: age, sex, BMI, WC, AN, and culture identity score, leaving a total n = 4141 (initial n = 5558). There were missing data for parent/caregiver income (n = 929 included in analysis as “not reported”), education (n = 12), and child race/ethnicity (n = 16). Table 1 shows the summary statistics for the participants. The majority of the children fell into the Healthy weight (70.7%), WC < 75th (71.3%) and <90th (88.3%) percentiles categories, and a great majority did not have AN (95.1%) (Table 1). Most of the parents/caregivers associated with a cultural identity score of integrated (70.1%), followed by traditional (23.5%), marginalized (3.7%), and assimilated (2.6%). Table 1 displays the inferential statistics for all the categorical variables and prevalence percentages of OWOB, >75th and >90th WC percentiles, and AN. The prevalence percentages of OWOB, WC > 75th and >90th percentiles ran about a third or less for all variables except for AIAN or Black (OWOB prevalence = 49.3% and WC > 75th percentile prevalence = 52.2%). The prevalence of AN was very low for all variable categories (under 14%) and there were no cases of reported AN for White, AIAN or Black, and children from Alaska. AN was highest among Asian children (8.7%). The outcomes in Table 1 varied by the categorical variables, as evidenced by the Chi-square tests.

Table 2 and Table 3 show logistic regression models. Model 1a, 2a, and 3a show the cultural identity score with OWOB, high WC, and presence of AN. A cultural identity of traditional and marginalized was inversely associated with OWOB, WC ≥ 75th and ≥90th percentiles (all were statistically significant except for marginalized for WC > 90th percentile). Table 3 shows the final presented models for all dependent variables. After adjusting for child sex, age, race, and ethnicity, parent/caregiver education, household language, household income, and multigenerational household status, the inverse associations of traditional identity with OWOB and WC ≥ 75th percentile remained significant. Marginalized cultural identity remained inversely associated with the outcomes after adjustment but were no longer significant.

## 4. Discussion

Although this study did not find an association between U.S. cultural identity (assimilated) and the outcome variables, the traditional and marginalized cultural identity categories were found to be inversely associated with OWOB compared to the integrated cultural identity category, even after adjustment. Participants with a high affinity for the traditional culture were inversely associated with WC ≥ 75th percentile compared to the integrated culture identity score, even after adjustment. Having close ethnicities and consuming traditional diets [34] have also been shown to have positive effects on health status. It is unclear why those (parents) with a lower affiliation with the U.S. mainland and with a traditional culture would have children with lower odds of OWOB. This could be driven by the FAS jurisdictions where traditional status is higher and OWOB is lower. Traditional lifestyles may include more local produce consumption and less sedentary behavior. However, conclusions are primarily for integrated and traditional group comparisons due to the small numbers associated with the marginalized and assimilated groups.

Aligned with some, but not all, of the literature, there were several consistent correlations of child health status and culture and the parent’s cultural identity. Parents/caregivers who had more than a high school education had higher odds of having children in the OWOB category, as well as WC ≥ 75th and ≥90th percentile categories compared to those with less than a high school education. This difference in socioeconomic status (SES), and perhaps greater household funds, could be the reason for this disparity. Language is an important indicator of cultural identity [35], and those who were multilingual had increased odds for OWOB and WC ≥ 75th percentile when compared to those who spoke English only. In adult studies, however, biculturalism (as indicated by language) has been shown to have a protective effect [36]. Other studies have revealed a positive association between Spanish fluency and obesity in U.S.-born Hispanics [37]. Novotny et al. showed that ethnically mixed children from the USAPI, Alaska, and Hawaiʻi have a higher risk for OWOB which may be related to having more access to foods from their respective cultures [18]. For OWOB status, being Asian, White, or more than one race (compared to NHPI) was protective. However, as stated above, mixed ethnicities have been shown to have a higher risk of OWOB in the limited literature from the USAPI, Alaska, and Hawaiʻi [18,38]. The odds of having a WC ≥ 75th percentile was decreased by 25% for children of more than one race. All jurisdictions (except Hawaiʻi and Alaska) had a protective effect against OWOB and WC > 75th and >90th percentile compared to American Samoa. American Samoa had the highest OWOB prevalence percentages (42.5%) within the USAPI, Alaska, and Hawaiʻi. Novotny et al. found similar results [18].

There is evidence that WC is a better indicator of cardiovascular (CV) disease [39] and visceral fat [40] than BMI because it is examining trunk adiposity. However, exact WC cut-off points to determine trunk adiposity risk are not as recognized compared with the BMI age- and sex-specific percentiles for children. Studies have used different cut-off points for WC to determine those children and adults at greater health risk [41]. A higher CV risk has been shown in pre-pubescent children with a WC > 90th percentile [42] and has been recommended to be used as a cut-off value for the screening of metabolic syndrome in children [43]. Another study among children aged 3–19 years suggested the 80th percentile in order to properly measure trunk adiposity [40]. Ethnic differences in WC distribution among children also need to be taken into account [41]. The use of 75th percentile and 90th percentile in this study was beneficial so those in ≥75th percentile would not be left out and could be seen as an “action point” [27] for (overweight) health initiatives, while more targeted efforts can be made for those that fall into the ≥90th percentile (obese) until proper universal cut-off points are established.

There are some limitations to be mentioned in this study. Firstly, WC measurements were taken at the level of the umbilicus because of its easy reference point and reduction of measurement error [21], while some other studies have measured WC between the top of the iliac crest and the last palpable rib in the mid-axillary line [27,39,44]. These discrepancies may impact comparisons with other studies. Nevertheless, there should not be a significant impact because the difference in measurement between the umbilicus and iliac in pre-pubescent children is minimal [45] and this study focuses on variation within the population, not prevalence of abdominal obesity. To further potential WC discrepancies, the WC percentiles were adjusted to classify ≥75th percentile as overweight and ≥90th percentile as obese in this study. This is not a universal cut-off point for WC which may impact comparisons with other studies. Secondly, there was also no significant association between AN and assimilation. While BMI and WC were measured metrically using standardized approaches, AN was assessed visually by examining the color and texture of the skin around the neck and may be more prone to misclassification. AN is harder to assess in lighter skinned participants. The assessment is based on the darkening of the skin, which may be more visible in darker individuals who have more melanin and may be more common in the USAPI, Alaska, and Hawaiʻi. The low numbers reported in this study may also be due to age at diagnosis. Thirdly, missing data and no dietary, physical activity, or sleep information used in this analysis could be residual confounding. However, these data are available and can be used in a future study. In addition, the unique and diverse mixed ethnic populations in the USAPI, Alaska, and Hawaiʻi made grouping of ethnicities for statistical analysis difficult and could have masked cultural differences, especially with current growth standards (WHO) and references (CDC) which do not account for these ethnic differences. Furthermore, a more comprehensive tool to assess cultural identity in future studies would be able to capture this variable more clearly. Lastly, due to the observational cross-sectional nature of this study, causality cannot be determined. Some strengths of this study include its large sample size and the novelty of this study, as there are extremely limited data on this area that looks at acculturation/enculturation and obesity among U.S.-Affiliated Pacific children.

## 5. Conclusions

There was no significant association between AN and acculturation/enculturation. Those parents/caregivers categorized in the traditional cultural identity category were found to have an inverse association with their children having a WC ≥ 75th percentile, even after adjustment. Also, those who were categorized as marginalized were more likely to have children with OWOB, even after adjustment. Further testing of interventions in the jursidictions across the USAPI, Alaska, and Hawaiʻi promoting a strong connection with traditional culture may be warranted to understand why it has a protective effect on the health of their children and to help reduce the health risks associated with non-communicable diseases that are on the rise in the region. Future studies should assess a complete profile of cultural practices and language(s) spoken (at home) as it influences OWOB risk factors like diet, sleep, and physical activity among these underrepresented groups of people in the USAPI, Alaska, and Hawaiʻi.

## Figures and Tables

**Table 1:** Characteristics of the participating children aged 2–8 years by Healthy weight and Overweight/Obese (OWOB) status, waist circumference (WC) percentile, and acanthosis nigricans (AN) distribution or prevalence (n = 4121).

	Total n (%)	Healthy Weight n (%)	OWOB n (%)	*p*-Value ^a^	WC < 75th n (%)	WC > 75th n (%)	*p*-Value ^a^	WC < 90th n (%)	WC > 90th n (%)	*p*-Value ^a^	No AN n (%)	Yes AN n (%)	*p*-Value ^a^
**All Participants**	4121 (100)	2913 (70.7)	1208 (29.3)	n/a	2939 (71.3)	1182 (28.7)	n/a	3640 (88.3)	481 (11.6)	n/a	3919 (95.1)	202 (4.9)	n/a
**Age [years, mean (SD)]**	[5.0 (1.6)]	[5.0 (1.6)]	[5.1 (1.7)]	0.3659	[5.2 (1.6)]	[4.7 (1.7)]	**<0.0001**	[5.1 (1.6)]	[4.6 (1.7)]	**<0.0001**	[5.0 (1.6)]	[5.7 (1.7)]	**<0.0001**
**Sex**													
Boy	2090 (50.7)	1439 (68.9)	651 (31.2)	**0.0087**	1447 (69.2)	643 (30.8)	**<0.0027**	1822 (87.2)	268 (12.8)	**0.0196**	1987 (95.1)	103 (4.9)	0.9363
Girl	2031 (49.3)	1474 (72.6)	557 (27.4)		1492 (73.5)	539 (26.5)		1818 (89.5)	213 (10.5)		1932 (95.1)	99 (4.9)	
**Cultural Identity Score**													
Integrated	2890 (70.1)	1993 (69.0)	897 (31.0)	**0.0004**	1985 (68.7)	905 (31.3)	**<0.0001**	2529 (87.5)	361 (12.5)	**0.0403**	2755 (95.3)	135 (4.7)	0.2605
Traditional	969 (23.5)	727 (75.0)	242 (25.0)		757 (78.1)	212 (21.9)		879 (90.7)	90 (9.3)		911 (94.0)	58 (6.0)	
Assimilated	108 (2.6)	72 (66.7)	36 (33.3)		78 (72.2)	30 (27.8)		93 (86.1)	15 (13.9)		104 (96.3)	4 (3.7)	
Marginalized	154 (3.7)	121 (78.6)	33 (21.4)		119 (77.3)	35 (22.7)		139 (90.3)	15 (9.7)		149 (96.8)	5 (3.3)	
**Parent/Caregiver Education**													
High school or lower	2551 (62.1)	1848 (72.4)	703 (27.6)	**0.0011**	1899 (74.4)	652 (25.6)	**<0.0001**	2283 (89.5)	268 (10.5)	**0.0027**	2420 (94.9)	131 (5.1)	0.4056
More than high school	1558 (37.9)	1054 (67.7)	504 (32.4)		1031 (66.2)	527 (33.8)		1346 (86.4)	212 (13.6)		1487 (95.4)	71 (4.6)	
**Household Income**													
Less than $35,000	2515 (61.0)	1742 (69.3)	773 (30.7)	**<0.001**	1763 (70.1)	752 (29.9)	**<0.0001**	2210 (87.9)	305 (12.1)	**<0.0001**	2383 (94.8)	132 (5.3)	**0.0006**
More than or equal to $35,000	677 (16.4)	452 (66.8)	225 (33.2)		435 (64.3)	242 (35.8)		573 (84.6)	104 (15.4)		663 (97.9)	14 (2.1)	
Not reported	929 (22.5)	719 (77.4)	210 (22.6)		741 (79.7)	188 (20.2)		857 (92.3)	72 (7.8)		873 (94.0)	56 (6.0)	
**Multigenerational Household**													
No	2642 (64.1)	1822 (69.0)	820 (31.0)	**0.0012**	1838 (69.6)	804 (30.4)	**0.0009**	2322 (87.9)	320 (12.1)	0.2396	2522 (95.5)	120 (4.5)	0.1529
Yes	1479 (35.9)	1091 (73.8)	388 (26.2)		1101 (74.4)	378 (25.6)		1318 (89.1)	161 (10.9)		1397 (94.5)	82 (5.5)	
**Race/Ethnicity**													
Asian	252 (6.1)	186 (73.8)	66 (26.2)	**0.004**	185 (73.4)	67 (26.6)	**<0.0001**	227 (90.1)	25 (9.9)	**0.0622**	230 (91.3)	22 (8.7)	**<0.0001**
More than one race	764 (18.6)	547 (71.6)	217 (28.4)		533 (69.8)	231 (30.2)		666 (87.2)	98 (12.8)		746 (97.6)	18 (2.4)	
NHPI	2777 (67.7)	1967 (70.8)	810 (29.2)		2030 (73.1)	747 (26.9)		2471 (89.0)	306 (11.0)		2616 (94.2)	161 (5.8)	
AIAN or Black	67 (1.6)	34 (50.8)	33 (49.3)		32 (47.8)	35 (52.2)		54 (80.6)	13 (19.4)		67 (100.0)	0 (0.0)	
White	245 (6.0)	166 (67.8)	79 (32.2)		148 (60.4)	97 (39.6)		209 (85.3)	36 (14.7)		245 (100.0)	0 (0.0)	
**Indigenous ^b^**													
No	896 (21.8)	614 (68.5)	282 (31.8)	0.1007	609 (68.0)	287 (32.0)	**0.0129**	782 (87.3)	114 (12.7)	0.2869	854 (95.3)	42 (4.7)	0.7177
Yes	3211 (78.2)	2291 (71.4)	920 (28.7)		2319 (77.2)	892 (27.8)		2844 (88.6)	367 (11.4)		3051 (95.0)	160 (5.0)	
**Language**													
English only	1635 (39.8)	1140 (69.7)	495 (30.3)	**<0.0001**	1127 (68.9)	508 (31.1)	**<0.0001**	1429 (87.4)	206 (12.6)	**<0.0001**	1592 (97.4)	43 (2.6)	**<0.0001**
No English	1383 (33.6)	1065 (77.0)	318 (23.0)		1090 (78.8)	293 (21.2)		1275 (92.2)	108 (7.8)		1296 (93.7)	87 (6.3)	
English and 1 or more other language(s)	1095 (26.6)	702 (64.1)	393 (35.9)		716 (65.4)	379 (34.6)		929 (84.8)	166 (15.2)		1023 (93.4)	72 (6.6)	
**Jurisdiction**													
Palau	173 (4.2)	130 (75.1)	43 (24.9)	**<0.0001**	132 (76.3)	41 (23.7)	**<0.0001**	160 (92.5)	13 (7.5)	**<0.0001**	162 (93.6)	11 (6.4)	**<0.0001**
Yap	172 (4.2)	152 (88.4)	20 (11.6)		133 (77.3)	39 (22.7)		157 (91.3)	15 (8.7)		167 (97.1)	5 (2.9)	
Guam	664 (16.1)	487 (73.3)	177 (26.7)		516 (77.7)	148 (22.3)		607 (91.4)	57 (8.6)		642 (96.7)	22 (3.3)	
CNMI	544 (13.2)	388 (71.3)	156 (28.7)		410 (75.4)	134 (24.6)		482 (88.6)	62 (11.4)		493 (90.6)	51 (9.4)	
Chuuk	152 (3.7)	139 (91.5)	13 (8.6)		145 (95.4)	7 (4.6)		151 (99.3)	1 (0.7)		146 (96.1)	6 (4.0)	
Pohnpei	164 (4.0)	127 (77.4)	37 (22.6)		138 (84.2)	26 (15.9)		159 (97.0)	5 (3.1)		142 (86.6)	22 (13.4)	
Kosrae	169 (4.1)	148 (87.6)	21 (12.4)		148 (87.6)	21 (12.4)		165 (97.6)	4 (2.4)		164 (97.0)	5 (3.0)	
RMI	192 (4.7)	187 (97.4)	5 (2.6)		188 (97.9)	4 (2.1)		191 (99.5)	1 (0.5)		172 (89.6)	20 (10.4)	
American Samoa	922 (22.4)	530 (57.5)	392 (42.5)		556 (60.3)	366 (39.7)		759 (82.3)	163 (17.7)		871 (94.5)	51 (5.5)	
Hawaiʻi	583 (14.2)	386 (66.2)	197 (33.8)		358 (61.4)	225 (38.6)		487 (83.5)	96 (16.5)		574 (98.5)	9 (1.5)	
Alaska	386 (9.4)	239 (61.9)	147 (38.1)		215 (55.7)	171 (44.3)		322 (83.4)	64 (16.6)		386 (100.0)	0 (0.0)	

Abbreviations: AIAN, American Indian/Alaska Native; CNMI, Commonwealth of the Northern Mariana Islands; NHPI, Native Hawaiian or Pacific Islander; RMI, Republic of the Marshall Islands. ^a^ *p*-value is for testing weight status (healthy weight and overweight/obese), waist circumference percentile (<75th and >75th percentile, <90th percentile and >90th percentile), and Acanthosis Nigricans differences; bold numbers refer to significant value *p* < 0.15 were left out of logistic regression models. ^b^ Indigenous refers to culturally distinctive ethnicities that originated from that jurisdiction.

**Table 2:** Crude logistic regression models showing associations of cultural identity with child overweight/obesity (OWOB) status, waist circumference (WC, >75th percentile and >90th percentile), or acanthosis nigricans (AN).

	OWOB	WC > 75th	WC > 90th	AN (Yes) ^a^
	OR (95% CI)	OR (95% CI)	OR (95% CI)	OR (95% CI)
**Cultural Identity Score**				
Traditional vs Integrated	0.74 (0.63–0.87) *	0.61 (0.52–0.73) *	0.72 (0.56–0.92) *	1.30 (0.95–1.78)
Assimilated vs Integrated	1.11 (0.74–1.67)	0.84 (0.55–1.30)	1.13 (0.65–1.97)	0.79 (0.29–2.16)
Marginalized vs. Integrated	0.61 (0.41–0.90) *	0.65 (0.44–0.95) *	0.76 (0.44–1.30)	0.69 (0.28–1.70)

^a^ Cultural identity score and acanthosis nigricans was not significant at *p* < 0.15 or in the crude model so it was left out of the final adjusted model. * Indicates 95% confidence interval that is statistically significant (does not include the null value of 1). Abbreviations: OWOB, overweight/obesity; WC, waist circumference; AN, acanthosis nigricans; CI, confidence interval; OR, odds ratio.

**Table 3:** Adjusted logistic regression models ^a^ showing the factors associated with child overweight/obesity (OWOB) status and waist circumference (WC) percentiles.

	OWOB	WC > 75th	WC > 90th
	OR (95% CI)	OR (95% CI)	OR (95% CI)
**Cultural Identity Score** Traditional vs. Integrated	0.86 (0.75–0.98) *	0.77 (0.64–0.92) *	0.91 (0.73–1.13)
Assimilated vs. Integrated	1.14 (0.82–1.59)	0.89 (0.57–1.39)	1.20 (0.63–2.27)
Marginalized vs. Integrated	0.67 (0.42–1.09)	0.69 (0.42–1.14)	0.84 (0.43–1.62)
**Age**	1.01 (0.97–1.06)	0.85 (0.81–0.90) *	0.83 (0.79–0.88) *
**Sex** Girl vs. Boy	0.84 (0.74–0.94) *	0.81 (0.69–0.96) *	0.80 (0.68–0.94) *
**Parent/Caregiver Education** >High School vs. ≤High School	1.12 (0.94–1.34)	1.22 (1.06–1.42) *	1.14 (0.86–1.51)
**Multigenerational Household** Yes vs. No	0.85 (0.73–1.00)	0.87 (0.75–1.01)	0.97 (0.78–1.21)
**Race/Ethnicity** Asian vs. NHPI	0.59 (0.48–0.73) *	0.67 (0.47–0.97) *	0.64 (0.41–0.99) *
More than one race vs. NHPI	0.83 (0.66–1.04)	0.92 (0.74–1.15)	0.92 (0.70–1.21)
AIAN or Black vs. NHPI	1.85 (1.03–3.34) *	2.06 (0.84–5.09)	1.38 (0.72–2.65)
White vs. NHPI	0.88 (0.55–1.40)	1.15 (0.66–1.98)	0.91 (0.43–1.94)
**Language** No English vs. English only	0.74 (0.60–0.90) *	0.73 (0.57–0.93) *	0.66 (0.52–0.84) *
English and 1 or more other language(s) vs. English only	1.35 (1.11–1.63) *	1.37 (1.09–1.72) *	1.39 (1.07–1.82) *
**Household Income** <$35K vs. ≥$35K	1.00 (0.81–1.23)	0.96 (0.82–1.13)	0.88 (0.66–1.18)
Not Reported vs. ≥$35K	0.71 (0.55–0.92) *	0.65 (0.51–0.81) *	0.58 (0.41–0.82) *

^a^ All models were adjusted for the jurisdiction and community and controlled for all variables included in the table. * Indicates 95% confidence interval that is statistically significant (does not include the null value of 1). Abbreviations: AIAN, American Indian/Alaska Native; CNMI, Commonwealth of the Northern Mariana Islands; CI, confidence interval; NHPI, Native Hawaiian or Pacific Islander; OR, odds ratio.

## Data Availability

Data described in the manuscript, codebook, and analytic code will be made available upon request (pending requirements, e.g., application, approval, payment).
